# Addressing workplace practices: combating the rising prevalence of computer vision syndrome

**DOI:** 10.1097/MS9.0000000000005204

**Published:** 2026-06-03

**Authors:** Syed Shahmeer Ahmed, Shakeeba Zubair, Nadia Mehmood, Muhammad Tauseef Haider Malik, Abedin Samadi

**Affiliations:** a Department of Medicine, Karachi Medical & Dental College, Karachi, Pakistan; b Department of Medicine, PNS Shifa Hospital, Karachi, Pakistan; c Department of Medicine, Aga Khan University Hospital, Karachi, Pakistan; d Department of Medicine, Dow University of Health Sciences, Karachi, Pakistan; e Department of Medicine, Kabul University of Medical Sciences Abu Ali Sina, Kabul, Afghanistan

**Keywords:** 20-20-20 rule, computer vision syndrome, digital eye strain, dry eye disease, ergonomics

## Abstract

Computer vision syndrome (CVS) is an increasingly prevalent occupational health concern associated with prolonged use of digital devices, affecting millions worldwide. Characterized by symptoms such as eyestrain, dryness, blurred vision, redness, and headaches, CVS significantly affects productivity and quality of life. This review highlights the growing burden, risk factors, pathophysiology, and preventive strategies related to CVS. Key contributing factors include extended screen time, poor ergonomics, reduced blink rate, improper screen positioning, and lack of regular breaks. Dry eye disease plays a central role in its pathogenesis due to increased corneal exposure and decreased tear film stability. Preventive strategies, particularly the 20-20-20 rule, have shown effectiveness in reducing symptoms and improving ocular surface health. Ergonomic modifications, such as positioning screens below eye level and maintaining proper posture, further help in minimizing discomfort. Despite its high prevalence, awareness regarding CVS remains inadequate among both employees and employers, leading to underutilization of preventive measures. Implementation of workplace policies, regular awareness sessions, and adherence to evidence-based ergonomic practices are essential to mitigate the rising burden of CVS. Addressing these factors is crucial to promote ocular health and ensure a safer, more productive working environment in the digital age.

1. **Growing global burden of computer vision syndrome**

Computer vision syndrome (CVS) is a common condition now. Global data suggest that 60 million people suffer from it. Moreover, a million new cases occur each year^[^1^]^. A number of ocular symptoms linked to computer use are becoming more and more common as computers become a part of daily life. These symptoms, which are collectively referred to as CVS, include eyestrain, fatigue, irritability, redness, blurred vision, and double vision^[^2^]^. CVS, one of the top workplace risks of the 21st century, can be brought on by continuous computer use for 3 hours or more each day^[^2^]^. CVS affects almost two out of every three individuals.

2. **Clinical features and risk factors of CVS**

Bad posture, using electronics outside of work, not taking breaks often, using visual display terminals for extended periods, using short-distance screens, and general ergonomic practices were all linked to an increased risk of developing CVS^[^3^]^.

3. **Dry eye disease as a central pathophysiological mechanism**

A major root cause of CVS has already been identified as dry eyes. As a result of the monitor regularly being held in the main gaze, increased corneal exposure and decreased blink rate and amplitude are likely to be the causes of dry eye complaints^[^4^]^.

4. **Preventive strategies: the 20-20-20 rule**

To reduce these dry eye symptoms, the 20-20-20 rule can be used, which was designed by Jeffrey Anshel, an optometrist. Clinicians usually advise taking regular breaks to reduce digital eye strain. The so-called 20-20-20 rule states that people should focus on something at least 20 feet (6 m) away for at least 20 seconds once every 20 minutes^[^5^]^. The 20-20-20 rule’s educational intervention results in considerable improvements in dry eye symptoms and tear film, as well as some modest improvements in ocular surface integrity^[^6^]^.

5. **Role of ergonomics and screen positioning**

If the gadget was kept slightly below eye level, ocular discomfort and neck pain were less likely to occur. University students frequently experience symptoms of CVS, which might be lessened with better ergonomic techniques. These symptoms include neck aches, eye strain, and burning^[^7^]^.

6. **Need for workplace awareness and policy implementation**

The workers and companies overlook or are not aware of the syndrome, and no preventive measures are taken by the workers themselves or the companies to prevent the worsening symptoms or the prevalence of this condition. Therefore, these practices should be implemented at every workplace to provide a safe, healthy, and comfortable environment. Moreover, the management needs to hold an awareness session to ensure that all workers follow the measures promptly.

To ensure methodological transparency and ethical compliance in manuscript preparation, we adhered to the recently developed TITAN (Transparency in the Reporting of Artificial Intelligence) 2025 guidelines, which outline standards for responsible AI use in biomedical writing and publishing^[^8^]^.

## Data Availability

Not applicable.

## References

[1] Zenbaba D Sahiledengle B Bonsa M. Prevalence of computer vision syndrome and associated factors among instructors in ethiopian universities: a web-based cross-sectional study. ScientificWorldJournal 2021;2021:3384332. 34650344 10.1155/2021/3384332 PMC8510801

[2] Blehm C Vishnu S Khattak A. Computer vision syndrome: a review. Surv Ophthalmol 2005;50:253–62. 15850814 10.1016/j.survophthal.2005.02.008

[3] Lema AK Anbesu EW. Computer vision syndrome and its determinants: a systematic review and meta-analysis. SAGE Open Med 2022;10:20503121221142402. 36518554 10.1177/20503121221142402 PMC9743027

[4] Rosenfield M. Computer vision syndrome: a review of ocular causes and potential treatments. Ophthalmic Physiol Opt 2011;31:502–15. 21480937 10.1111/j.1475-1313.2011.00834.x

[5] Johnson S Rosenfield M. 20-20-20 rule: are these numbers justified? Optom Vis Sci 2023;100:52–56. 36473088 10.1097/OPX.0000000000001971

[6] Alabdulkader B. Effect of digital device use during COVID-19 on digital eye strain. Clin Exp Optom 2021;104:698–704. 33689614 10.1080/08164622.2021.1878843

[7] Mowatt L Gordon C Santosh ABR. Computer vision syndrome and ergonomic practices among undergraduate university students. Int J Clin Pract 2018;72:e13035. 10.1111/ijcp.13035 28980750

[8] Agha RA Mathew G Rashid R. TITAN Group. Transparency in the reporting of artificial intelligence—the TITAN guideline. Prem J Sci 2025;10: 100082

